# A Safe Maritime Path Planning Fusion Algorithm for USVs Based on Reinforcement Learning A* and LSTM-Enhanced DWA

**DOI:** 10.3390/s26030776

**Published:** 2026-01-23

**Authors:** Zhenxing Zhang, Qiujie Wang, Xiaohui Wang, Mingkun Feng

**Affiliations:** 1 School of Computer Science and Technology, Zhejiang University of Science and Technology, Hangzhou 310023, China; 2 School of Artificial Intelligence and Information Engineering, Zhejiang University of Science and Technology, Hangzhou 310023, China; 222308855049@zust.edu.cn (Q.W.); 222408855031@zust.edu.cn (X.W.); mkfeng96@zust.edu.cn (M.F.)

**Keywords:** path planning, dynamic window approach, A*, reinforcement learning, LSTM

## Abstract

In complex maritime environments, the safety of path planning for Unmanned Surface Vehicles (USVs) remains a significant challenge. Existing methods for handling dynamic obstacles often suffer from inadequate predictability and generate non-smooth trajectories. To address these issues, this paper proposes a reliable hybrid path planning approach that integrates a reinforcement learning-enhanced A* algorithm with an improved Dynamic Window Approach (DWA). Specifically, the A* algorithm is augmented by incorporating a dynamic five-neighborhood search mechanism, a reinforcement learning-based adaptive weighting strategy, and a path post-optimization procedure. These enhancements collectively shorten the path length and significantly improve trajectory smoothness. While ensuring that the global path avoids dynamic obstacles smoothly, a Kalman Filter (KF) is integrated into the Long Short-Term Memory (LSTM) network to preprocess historical data. This mechanism suppresses transient outliers and stabilizes the trajectory prediction of dynamic obstacles. Moreover, the evaluation function of the DWA is refined by incorporating the International Regulations for Preventing Collisions at Sea (COLREGs) constraints, enabling compliant navigation behaviors. Simulation results in MATLAB demonstrate that the enhanced A* algorithm better conforms to the kinematic model of the USVs. The improved DWA significantly reduces collision risks, thereby ensuring safer navigation in dynamic marine environments.

## 1. Introduction

Autonomous systems have become increasingly prevalent in modern maritime applications. As a low-cost and highly maneuverable maritime vehicle, USVs can adapt to most conditions at sea, and trajectory optimization in maritime networks has become a key research focus [1]. Empowered by intelligent algorithms [2], they are capable of performing tasks autonomously under various circumstances. However, safety concerns remain in autonomous path planning for public waters and narrow channels. In the complex and ever-changing marine environment [3], USVs may encounter numerous situations during navigation. Any delays in response or suboptimal path planning can compromise both the safety and efficiency of USVs operations at sea.

The path planning methods for USVs are primarily divided into global path planning and local path planning. Global path planning mainly relies on prior maps, while local path planning depends on obstacle information collected by sensors. Due to the variable and complex maritime conditions [4], it is usually necessary to integrate both global and local path planning to address these challenges.

In global path planning, as summarized by Lixing Liu et al. [5], commonly used algorithms include Dijkstra, A*, RRT, and the particle swarm algorithm, among others. The principles and applicable environments of these algorithms differ. Recent studies have also explored automated path planning strategies for robotic inspection in complex infrastructure environments, providing valuable insights for maritime applications [6]. Comparing these algorithms, A* balances efficiency and optimality. It can find the optimal solution based on a heuristic function and reduces the number of unnecessary nodes to explore by guiding the search direction towards the goal. However, the A* algorithm still has some issues. For instance, in areas close to obstacles, it may produce paths with multiple right-angle turns, resulting in unsmooth trajectories. Many scholars have made improvements and optimizations to the A* algorithm. Yuanmin Liu et al. [7] proposed a time-optimal A* algorithm which enhances the optimal path of the A* algorithm by modifying the heuristic method based on an analytical quantification of time cost. Similarly, Zhi Lin et al. [8] integrated prejudgment planning strategy and redundant inflection point elimination strategy into A-star to improve the quality of the path. The principle of enhancement, drawing inspiration from A*’s heuristic-driven search, has also been applied to a wide range of nature-inspired path planning algorithms. For example, Yujie Huang et al. [9] improved the whale optimization algorithm by incorporating segment learning and adaptive operator selection, which enhanced the algorithm’s search capability. Similarly, Long et al. [10] proposed an improved particle swarm optimization with neighbor adjustment strategies to enhance task scheduling efficiency, which shares similarities with neighborhood search optimization in path planning. In addition, machine learning algorithms have also been employed for path planning. Xiaobing Yu et al. [11] proposed a reinforcement learning-based multi-strategy cuckoo search algorithm. Furthermore, Ren et al. [12] explored multi-agent reinforcement learning with explicit neighborhood backtracking for network-wide control, while Gu and Wang [13] developed a constrained reinforcement learning approach to handle cooperative control in dense obstacle environments, demonstrating the efficacy of RL in complex constraints.

In dynamic environments, conventional collision avoidance algorithms such as Artificial Potential Field (APF) [14], DWA [15,16], and D* are commonly employed. These algorithms offer advantages such as fast computation and high real-time performance. Specifically, the DWA algorithm can effectively incorporate the kinematic constraints of the vehicle to generate rapid responses, making it widely applicable for local obstacle avoidance in various robotic and intelligent systems. However, a key limitation of DWA is that it only evaluates trajectories within a short simulation time window and lacks the capability for complex prediction of dynamic obstacles’ future intentions. To address these shortcomings, numerous researchers have proposed improvements. For instance, Dhong Hun Lee et al. [17] estimated the spatial distribution of obstacles using a Flow Map Field and predicted their future distributions to enhance the obstacle avoidance capability of DWA. Similarly, Hui Chen et al. [18] developed a decision tree-based DWA algorithm that adaptively adjusts its weighting parameters. Furthermore, Chuanbo Wu et al. have integrated machine learning methods with DWA to optimize its reward function, enabling the system to make optimal collision avoidance decisions in complex environments.

In summary, only by integrating global and local path planning [19,20] can a USV achieve a fully autonomous closed-loop navigation route. To address the limitations of the A* and DWA algorithms, we propose a global path optimization and predictive obstacle-avoidance algorithm. The specific improvements are as follows:

1. The heuristic function of the A* algorithm is adaptively optimized using a reinforcement learning approach to improve path quality.
2. A dynamic five-neighborhood search range is adopted to enhance the algorithm’s search speed.
3. An LSTM network, integrated with KF, is employed to predict the trajectories of dynamic obstacles. Enabling the LSTM to provide stable, accurate, and early judgments of obstacle movements to mitigate collision risks.
4. The evaluation function of the DWA is reformulated to strictly incorporate International Regulations for Preventing Collisions at Sea. LSTM enables the USVs to execute anticipatory and compliant avoidance maneuvers.

## 2. Environmental Map

**Figure 1:**
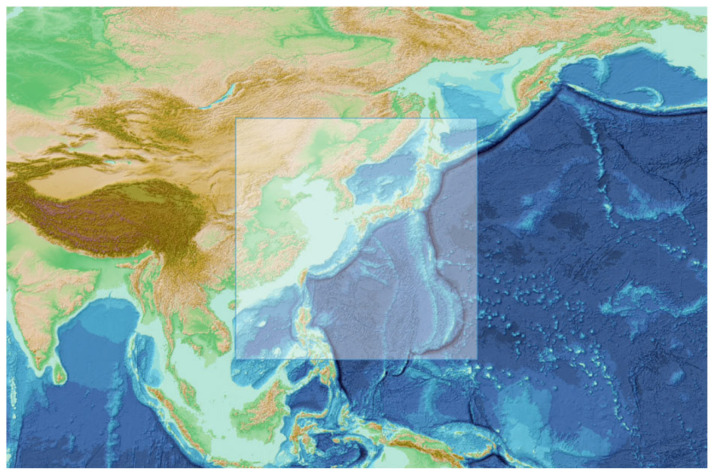
Achievable coverage area for grid-based mapping.

This study employs a grid-based method to model the continuous marine environment. The bathymetric data used for modeling is sourced from GEBCO [21]. By discretizing the continuous environment into regular grids for computational convenience, the algorithm extracts and imports the elevation map model. The overall area (The box-selected area in Figure 1) is defined within the coordinates (100° E and 10° N) to (150° E and 50° N), and the converted output grid file can be generated. The input parameters of the interface can be adjusted to modify the operational area for specific tasks. Experimental studies were conducted in multiple sea areas, yielding distinct results. In the grid, individual points are expanded multi-dimensionally to form impassable obstacle zones, significantly reducing the potential for contact with the vessel hull.

The data utilizes elevation data in the form of a latitude–longitude grid, with a longitude/latitude error of 0.0042 degrees. The USVs model is now initialized and transformed to be compatible with this grid model’s data.

## 3. Reinforcement A*

### 3.1. Conventional A*

The conventional A* algorithm [22] utilizes a heuristic function to estimate the cost from the current node to the goal node, thereby guiding the search direction, reducing the search space, and improving search efficiency [23]. By integrating the completeness of Dijkstra’s algorithm [24] with the directional advantages of both Breadth-First Search (BFS) and Greedy Best-First Search (GBFS), the A* algorithm achieves a unique hybrid mechanism. This enables the algorithm not only to find near-optimal paths but also to significantly enhance search efficiency, making it widely applicable in various fields.

The evaluation function for a node in the A* algorithm is defined as shown in Equation (1):

(1) f(n)=g(n)+h(n)

where

- g(n): the actual cost from the start node to the current node n;
- h(n): the heuristic estimated cost from the current node n to the goal node.

The key to the A* algorithm lies in the design of the heuristic function. Ideally, h(n) should accurately reflect the true cost from the node to the goal. In practical applications, the A* algorithm typically uses the Manhattan distance, Euclidean distance, or Chebyshev distance [25] to approximate h(n).

(2) $$h(n)=|x_{2}-x_{1}|+|y_{2}-y_{1}|$$

(3) $$h(n)=\sqrt{{\left(x_{2}-x_{1}\right)}^{2}+{\left(y_{2}-y_{1}\right)}^{2}}$$

(4) $$h(n)=\max(|x_{2}-x_{1}|,|y_{2}-y_{1}|)$$

Equations (2)–(4), respectively, express the Manhattan distance, Euclidean distance, and Chebyshev distance. $\left(x_{1},x_{2}\right)$ and $\left(y_{1},y_{2}\right)$ represent the coordinate values of the current node and the target node.

### 3.2. Q-Learning

The Q-learning algorithm is a classic reinforcement learning method belonging to the category of model-free, off-policy control approaches. This algorithm can be applied to any Markov Decision Process (MDP) to approximate an optimal policy [26]. The core of Q-learning lies in its use of temporal difference updates, where iterative estimations are employed to optimize the action-value function. In path planning tasks, a robot selects and executes an action based on the current environmental state, and the environment returns a corresponding reward while transitioning to a new state. If the robot receives a positive reward (+r), it becomes more likely to choose the same path again in the future; conversely, a negative reward (−r) reduces this tendency [27]. Through continuous feedback, the algorithm progressively refines its strategy over multiple interactions, optimizing decision-making until the policy converges or the task is completed.

(5) $$r_{t}=r(s_{t},a_{t},s_{t+1})$$

where

- $r_{t}$: the immediate reward function;
- $s_{t}$: the environmental state at the current time step;
- $a_{t}$: the action selected under state $s_{t}$;
- $s_{t+1}$: the new state reached after executing action $a_{t}$;

As shown in Equation (5), the probability distribution is determined by the initial state-action estimate at time step t, while the update rule for the optimal value function obtained through iterative computations is defined by the following equation:

(6) $$Q_{t+1}(s_{t},a_{t})=r_{t}+\gamma \underset{a_{t}}{\max}\{Q(s_{t+1},a_{t})|a_{t}\in A\}$$

where

- $Q_{t+1}(s_{t},a_{t})$: the updated Q-value after taking action $a_{t}$, in state $s_{t}$, at time t + 1;
- γ: the discount factor, which balances the importance of immediate rewards and future returns;
- $\underset{a_{t}}{\max}\{Q(s_{t+1},a_{t})$: the maximum Q-value among all possible actions in the next state $s_{t+1}$, and $a_{t}\in A$ signifies that the action $a_{t}$ belongs to the set of valid actions in the action space A.

When integrating the Q-learning with the A* algorithm, the system continuously selects actions according to ε−greedy policy during each episode to choose optimal actions, achieving learning objectives through iterative optimization of Q-values [28]. Furthermore, as the algorithm undergoes repeated learning, its exploration coefficient gradually decreases with extended training time, thereby maximizing the selection of optimal actions.

### 3.3. Improved A*

#### 3.3.1. Dynamic Five-Neighborhood Search

The conventional A* algorithm typically employs a four-neighborhood search strategy, considering only four possible movement directions on a two-dimensional plane. To enhance its performance, an improved version introduces an eight-neighborhood (eight-way) search. However, as the eight-way approach often generates redundant nodes, a dynamic five-neighborhood search concept is further incorporated. This refinement effectively reduces redundant nodes during the search process, thereby improving the algorithm’s operational speed and efficiency.

The dynamic five-neighborhood search adaptively adjusts the search direction in real-time, based on the relative positional relationship between the current node and the target point. The specific implementation involves selecting five prioritized expansion directions according to the angle θ between the current node and the target. This strategy not only maintains the completeness of the search but also significantly enhances search efficiency.

When obstacles are detected in all five prioritized neighborhood directions, the algorithm automatically switches to a full eight-neighborhood search to prevent deadlock situations. This adaptive adjustment mechanism ensures the algorithm’s reliability across diverse environments.

**Figure 2:**
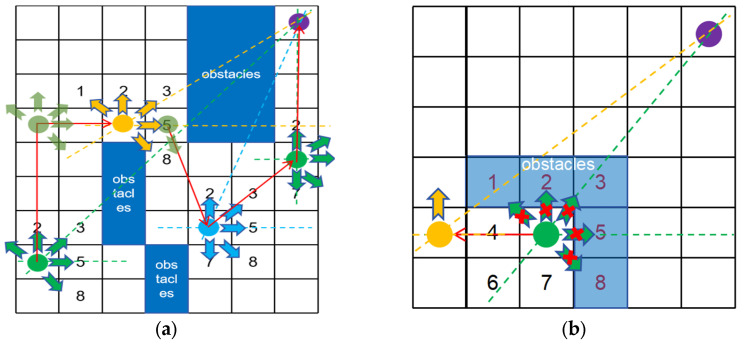
Dynamic Five-Neighborhood Search: (**a**) Schematic diagram of the five-neighborhood search path; (**b**) case of an inaccessible path using five-neighborhood search.

As illustrated in Figure 2, the dynamic five-neighborhood search method (Figure 2a) selects five prioritized directions based on the angle θ between the current node and the target. If an unreachable path is encountered (Figure 2b), the algorithm re-selects and explores the previously discarded nodes. The specific node indexing is detailed in Table 1 below:

#### 3.3.2. Reinforcement Learning-Based Adaptive Weighting

The dynamically weighted reinforcement learning algorithm employs an innovative hybrid architecture—the QL-A* algorithm—which integrates the global path planning capability of the conventional A* algorithm with the local optimization ability of Q-learning. By emphasizing real-time dynamic adjustment of the heuristic function, the algorithm continuously learns environmental features during the path planning process and progressively optimizes decision-making strategies. Compared with conventional methods, the improved algorithm demonstrates significant enhancements in path quality, search efficiency, and real-time performance.

The heuristic function is defined as follows:

(7) $$h_{enhanced}(n)=h_{base}(n)+\lambda_{p}\cdot P(n)+\lambda_{Q}\cdot Q(n)$$

where

- $h_{enhanced}(n)$: the value of the enhanced heuristic function, which integrates the conventional heuristic, potential field guidance, and learned experience;
- $h_{base}$: the base heuristic function;
- P(n): the potential field value, generated based on the attraction from the target point to guide the path toward convergence with the goal;
- Q(n): the action-value function learned through Q-learning, accumulating historical decision-making experience;
- $\lambda_{p}$: the weight coefficient for the potential field, controlling the strength of its guidance;
- $\lambda_{Q}$: the weight coefficient for the Q-value, adjusting the influence of learned experience.

Design of a Multi-Objective optimization reward function framework:

(8) $$r_{t}=w_{1}\cdot r_{distance}+w_{2}\cdot r_{progress}+w_{3}\cdot r_{smooth}+w_{4}\cdot r_{potential}$$

where

- $w_{1}$, $w_{2}$, $w_{3}$, and $w_{4}$: the weight coefficients for each component, namely basic movement cost, progress-toward-goal reward, smoothness penalty, and potential field adjustment, all satisfying the normalization condition $\sum w_{i}=1$.

Table 2 provides a detailed description of each component.

The algorithm employs a lightweight Q-table structure to facilitate experience accumulation across search cycles, with Q-value updates following standard reinforcement learning rules. As shown in Table 2, the reward function is designed to incorporate multiple factors, including movement distance cost, progress reward toward the goal, a slight penalty for diagonal movements, and an adjustment term for potential field variations.

**Figure 3:**
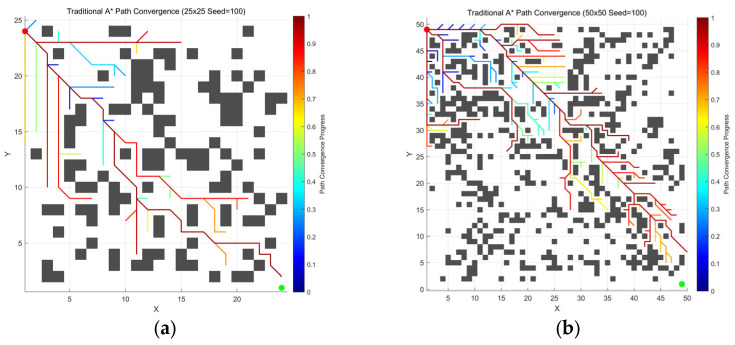

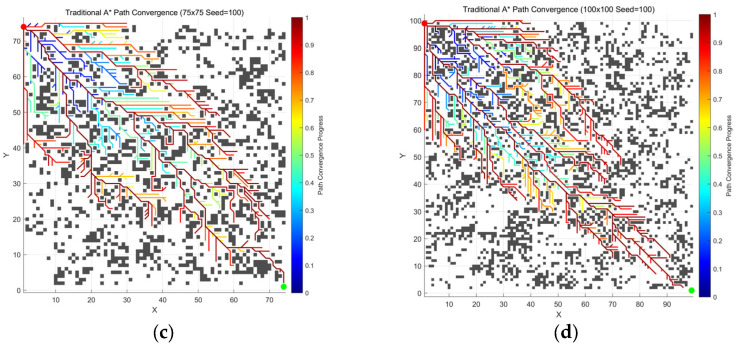
Convergence process of the conventional A* across environments of different scales: (**a**) 25 × 25 grid; (**b**) 50 × 50 grid; (**c**) 75 × 75 grid; (**d**) 100 × 100 grid.

**Figure 4:**
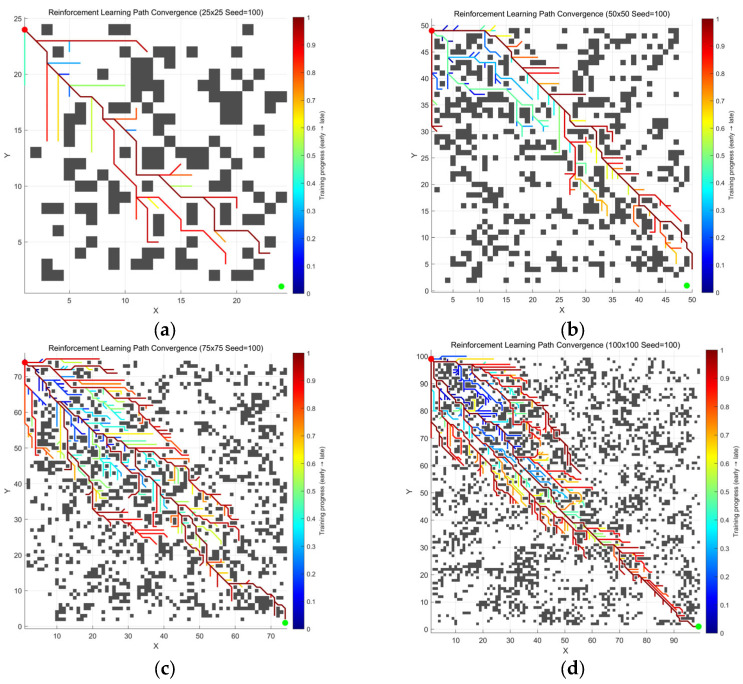
Convergence process of the reinforcement learning A* algorithm across environments of different scales: (**a**) 25 × 25 grid; (**b**) 50 × 50 grid; (**c**) 75 × 75 grid; (**d**) 100 × 100 grid.

Compared to the conventional A* (Figure 3), the enhanced A* algorithm by leveraging reinforcement learning (Figure 4) exhibits a more efficient search strategy, culminating in a notable decrease in the number of nodes expanded and a higher success rate in directional advancement toward the goal point.

#### 3.3.3. Path Optimization

The initial path generated by the improved A* algorithm still exhibits issues of redundant nodes and excessive turns. To ensure the stability and safety of the navigation system, a path optimization algorithm is employed for path post-processing.

**Figure 5:**
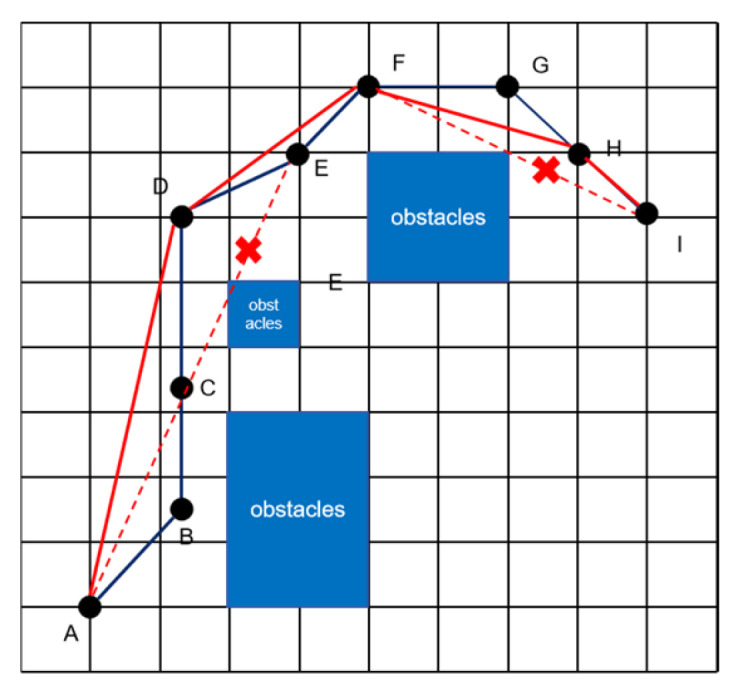
Path optimization processing. Path optimization processing. The black line indicates the originally generated path. The solid red line shows the final optimized path after smoothing. The red dashed lines represent invalid shortcut attempts that were rejected due to obstacle intersection.

As shown in Figure 5, taking the typical path A → I as an example, the algorithm sequentially evaluates the feasibility of direct connections between adjacent nodes. It checks whether non-consecutive nodes (e.g., A and C, A and D, etc.) can be connected via a straight line without intersecting obstacles. When certain intermediate nodes (e.g., B, C, etc.) are deemed skippable, the system directly connects feasible nodes (e.g., A to D), thereby eliminating redundant nodes.

Additionally, the algorithm incorporates a safety distance threshold. Even if no direct collision with obstacles occurs after removing redundant nodes, a path is still considered invalid if it passes too close to an obstacle (e.g., the direct connection from F to I is too near an obstacle). Through this systematic evaluation and optimization, unnecessary turning points are eliminated, significantly enhancing path continuity and motion efficiency while ensuring the path remains obstacle-free, and navigation reliability is maintained.

#### 3.3.4. Simulation Experiments

The simulation experiments were conducted on the MATLABR2025a platform with a comprehensive testing scheme. Four different map sizes—25 × 25, 50 × 50, 75 × 75, and 100 × 100—were selected for evaluation. For each map size, three distinct random seeds (Seed = 1, Seed = 100, and Seed = 1000) were used to generate randomized environments, ensuring the statistical significance of the test results.

The experiments compared the performance of the conventional A* algorithm and the improved A* algorithm across multiple test scenarios. In the results, the blue curve represents the path planning trajectory of the conventional A* algorithm, while the red curve corresponds to the planning results of the improved A* algorithm.

**Figure 6:**
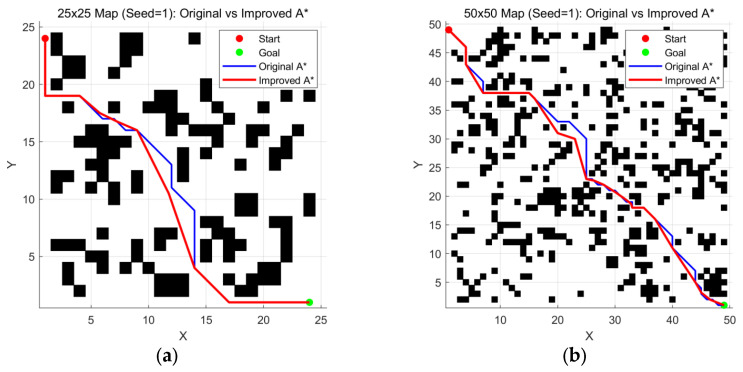

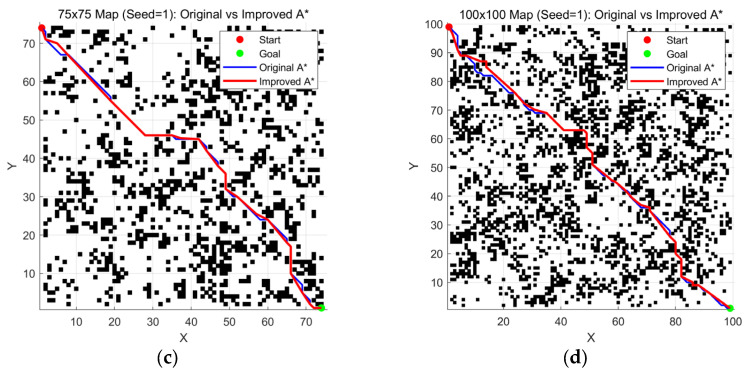
Comparison of maps generated with Seed = 1 at various scales: (**a**) 25 × 25 map; (**b**) 50 × 50 map; (**c**) 75 × 75 map; (**d**) 100 × 100 map.

As shown in Figure 6 and Table 3, data comparing Conventional A* and Improved A* indicate that on maps with seed = 1, Improved A* achieves significant improvements in path quality.

**Figure 7:**
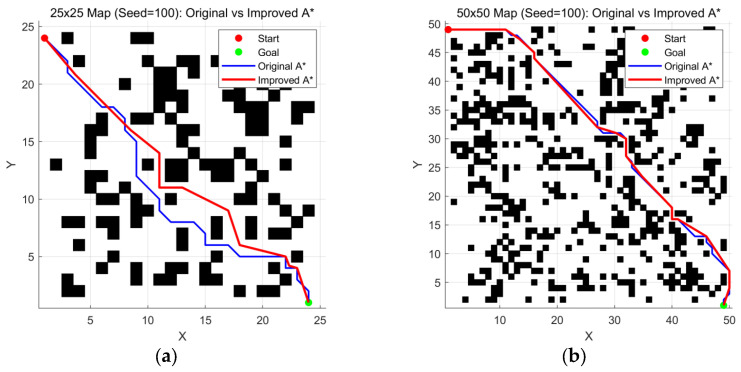

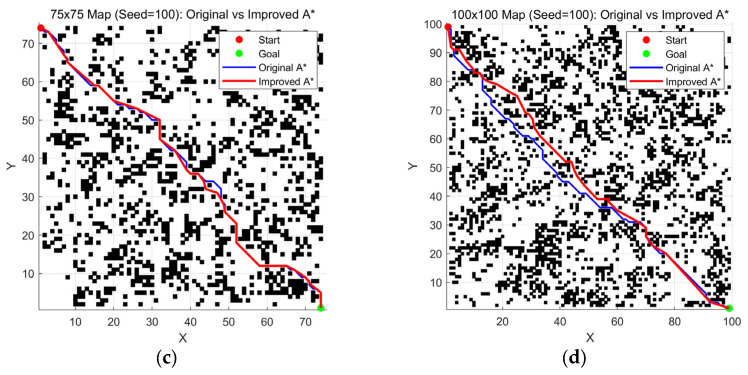
Comparison of maps generated with Seed = 100 at various scales: (**a**) 25 × 25 map; (**b**) 50 × 50 map; (**c**) 75 × 75 map; (**d**) 100 × 100 map.

As shown in Figure 7 and Table 4, the data comparison sustains the superiority of the improved A*, while the quality is maintained at the same level as when the seed = 1.

**Figure 8:**
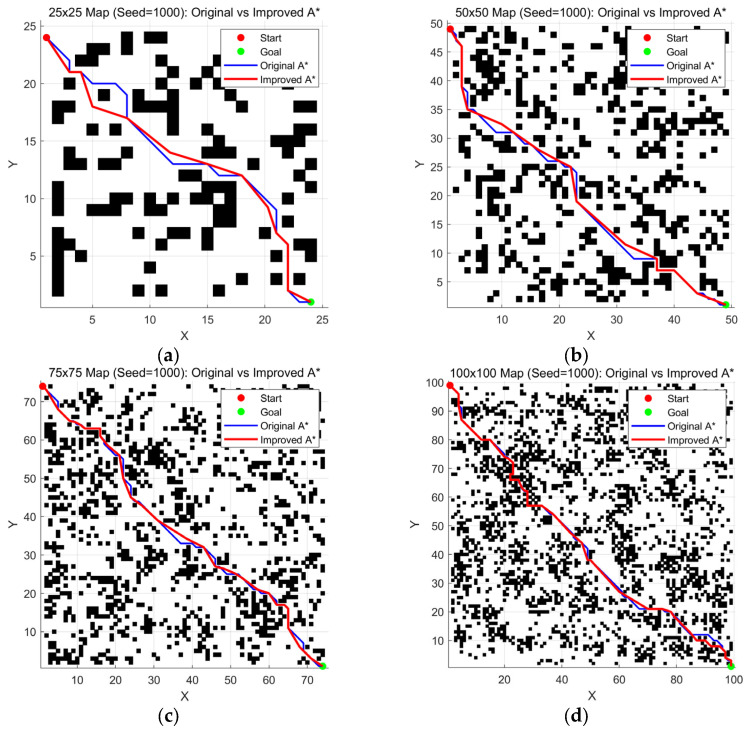
Comparison of maps generated with Seed = 1000 at various scales: (**a**) 25 × 25 map; (**b**) 50 × 50 map; (**c**) 75 × 75 map; (**d**) 100 × 100 map.

The experimental results in Figure 6, Figure 7 and Figure 8 and Table 3, Table 4 and Table 5 demonstrate that the improved A* algorithm significantly outperforms the conventional A* algorithm across all performance metrics. Specifically, paths generated by the improved algorithm are on average 5–8% shorter, have 30–45% fewer turning points, and are 50–70% smoother. Additionally, the number of expanded nodes is reduced by around 25–35%. These results conclusively demonstrate the comprehensive advantages of the improved algorithm in terms of path quality, search efficiency, and motion smoothness.

## 4. Improved DWA

### 4.1. Kinematic Model

In the DWA algorithm, establishing the motion model is a necessary step for initialization. The establishment of the motion model determines the determination of the sampling space in the DWA algorithm, thereby constraining the range of the motion space. The kinematic model and dynamic window for the next time step are as follows:

(9) $$v=\{v\in [v_{\min},v_{\max}],\omega \in [\omega_{\min},\omega_{\max}]\}$$

(10) $$v_{d}=\{(v_{t+1},\omega_{t+1})|v_{t+1}\in [v_{t}-a_{\max}\Delta t,v_{t}+a_{\max}\Delta t],\omega_{t+1}\in [\omega_{t}-\alpha_{\max}\Delta t,\omega_{t}+\alpha_{\max}\Delta t]\}$$

The updated position must satisfy the basic requirement of obstacle avoidance. Specifically, the Euclidean distance between the updated position and all obstacles must be greater than or equal to the sum of the USV’s minimum braking distance and the influence radius of the nearest obstacle.

(11) $$\left\{\begin{array}{c} x_{t+1}=x_{t}+v_{t+1}\cos\theta \cdot \Delta t\\ y_{t+1}=y_{t}+v_{t+1}\sin\theta \cdot \Delta t\\ \theta_{t+1}=\theta_{t}+\omega_{t+1}\cdot \Delta t \end{array}\right\}$$

(12) $$d((x_{t+1},y_{t+1}),obs)\geq r_{obs}+\frac{1}{2}\frac{v_{t+1}^{2}}{a_{\max}}$$

where

- d: minimum safe braking distance
- $\omega_{goal}$, $\omega_{obs}$: obstacle influence radius
- $a_{\max}$: maximum deceleration

### 4.2. LSTM

**Figure 9:**
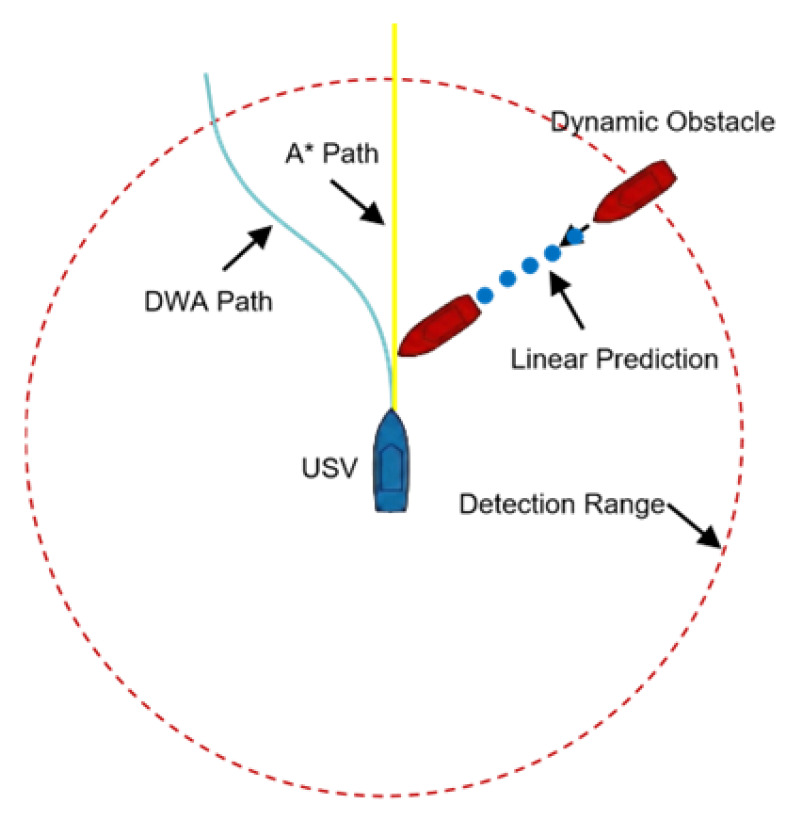
Simulation of conventional DWA.

In the DWA algorithm, typically only the vehicle’s own trajectory for the next step is simulated, without predicting the motion trajectories of dynamic obstacles. Consequently, it has weak predictive capability regarding dynamic obstacles and cannot formulate correct steering strategies. As illustrated in Figure 9, if the oncoming vessel is too fast, a collision may occur, posing a high risk. Moreover, COLREGs Rule 15 requires the give-way vessel to avoid crossing ahead of the other vessel.

Accurate prediction is crucial for safe motion planning; for instance, Liang et al. [29] utilized Transformer-based approaches for interaction-aware trajectory prediction in autonomous driving. To capture long-term dependencies in sequence data, advanced architectures like deep residual LSTM networks (ResLNet) have also been proposed [30]. In this study, a sequential prediction model is constructed based on the LSTM network to predict the motion trajectories of dynamic obstacles.

The model takes the incremental changes in historical consecutive positional coordinates as input, with the objective of predicting the positional increments at the next time step, thereby achieving autoregressive prediction of path trends.

**Figure 10:**
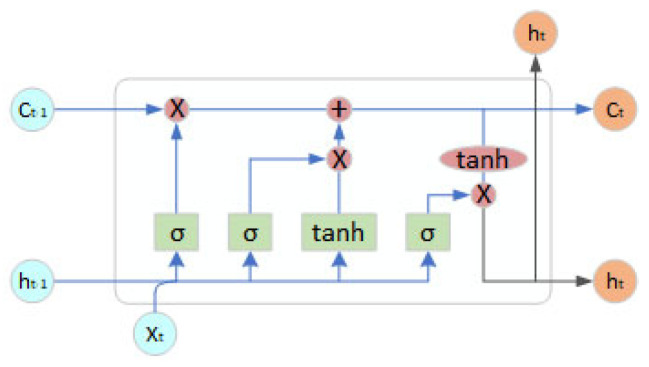
LSTM cell.

The LSTM [31] network is a type of recurrent neural network in which hidden nodes are replaced by LSTM cells (Figure 10). These cells are structured into three gates: the forget gate, the input gate, and the output gate. They are designed to carry relevant information across multiple time steps, avoiding the risks of vanishing or exploding gradients.

**Figure 11:**
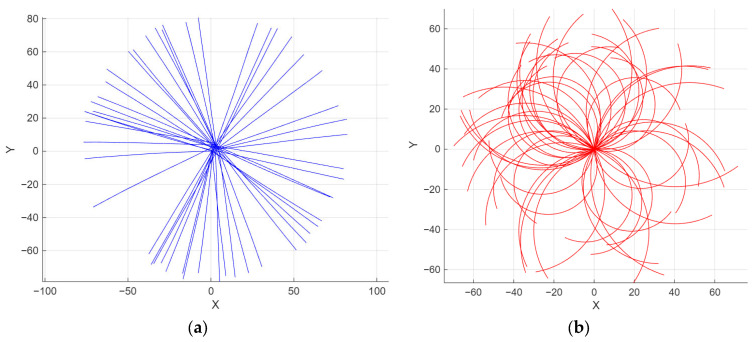
LSTM training kinematic simulator. (**a**) Linear trajectories; (**b**) multi−radius curvilinear trajectories.

The training data in Figure 11 originates from a kinematic simulator designed to replicate obstacle trajectories during autonomous navigation. To emulate real-world conditions, each path ba is subjected to randomized parametric variations, including velocity scaling and heading drift. The detailed training parameters configurations utilized for the LSTM model are presented in Table 6.

**Figure 12:**
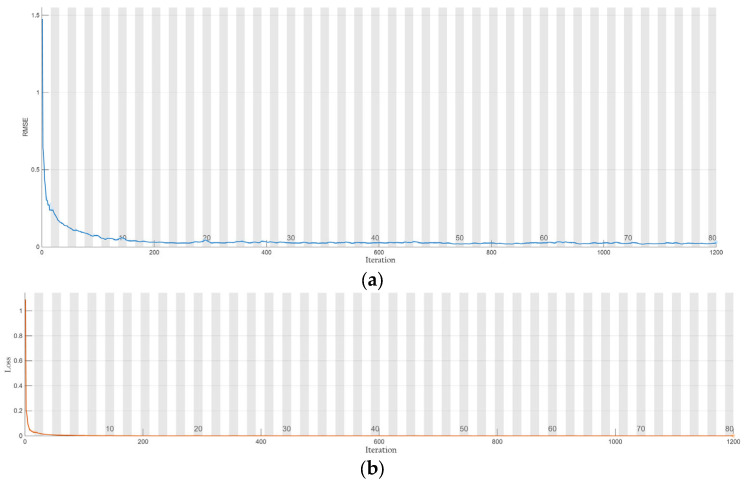
Training process of the model. (**a**) RMSE; (**b**) loss.

The training evolution of the LSTM architecture, illustrated in Figure 12, demonstrates robust convergence characteristics and high numerical precision. The Root Mean Square Error (RMSE) exhibits a rapid decay, stabilizing at approximately 200 epochs. Simultaneously, the loss function plateaus at a negligible magnitude, indicating that the network has successfully minimized the discrepancy between the predicted trajectories and the ground truth without manifesting overfitting.

**Figure 13:**
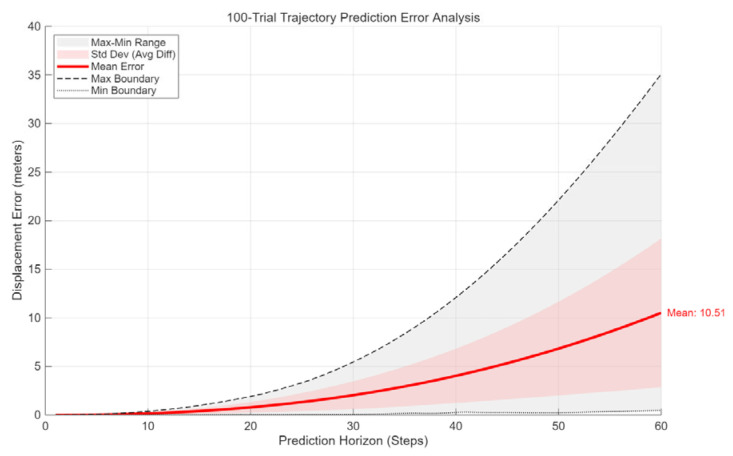
LSTM prediction performance.

To assess the model’s generalization capability, we conducted a closed-loop validation utilizing 100 newly generated trajectories, comprising both linear segments and curvilinear maneuvers with steering angles restricted to less than 20°. A comparative analysis between the LSTM-predicted trajectories and the ground truth-summarized in Figure 13 and Table 7 reveals high predictive fidelity within the initial 20-step horizon. However, as the temporal displacement increases, a cumulative divergence becomes evident. Notably, even within the intermediate 10-to-20 step interval, the maximum error metrics exhibit sporadic deviations. To mitigate prediction errors caused by sensor noise and enhance systemic robustness, a Kalman Filter [32] was employed as a dual-stage optimizer: first, to denoise the initial trajectory inputs, and subsequently, to recursively adjust the LSTM-generated outputs against the vehicle’s kinematic constraints.

To evaluate the impact of KF as a front-end trajectory denoising module on the performance of an LSTM-based trajectory prediction model, comparative experiments were conducted under different motion patterns, including straight-line motion and turning maneuvers.

**Figure 14:**
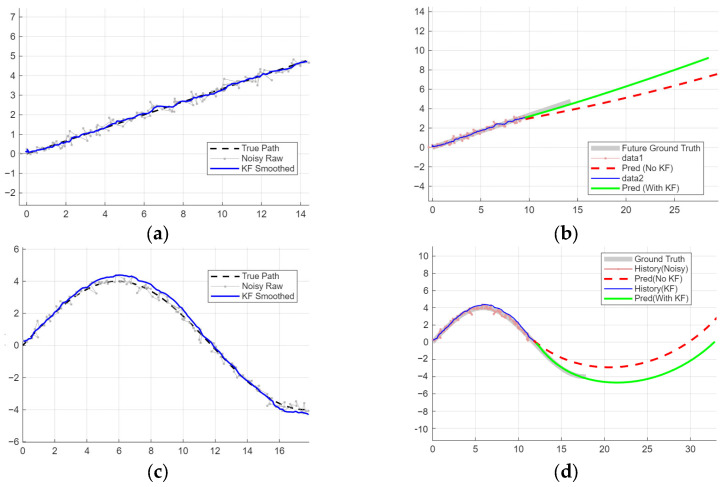
Comparison of path prediction performance using LSTM and KF. (**a**) Straight trajectories processed by KF; (**b**) straight trajectories predicted by LSTM with KF; (**c**) turning trajectories processed by KF; (**d**) turning trajectories predicted by LSTM with KF.

As presented in Figure 14, the predictions based on known path data exhibit reliable regularity and trajectory stability. The KF performs robustly in the data preprocessing stage (Figure 14a,c) and demonstrates a superior ability to smooth and refine the LSTM output trajectories (Figure 14b,d), ensuring more accurate and stable motion trends.

### 4.3. Adjustment of the Evaluation Function

**Figure 15:**
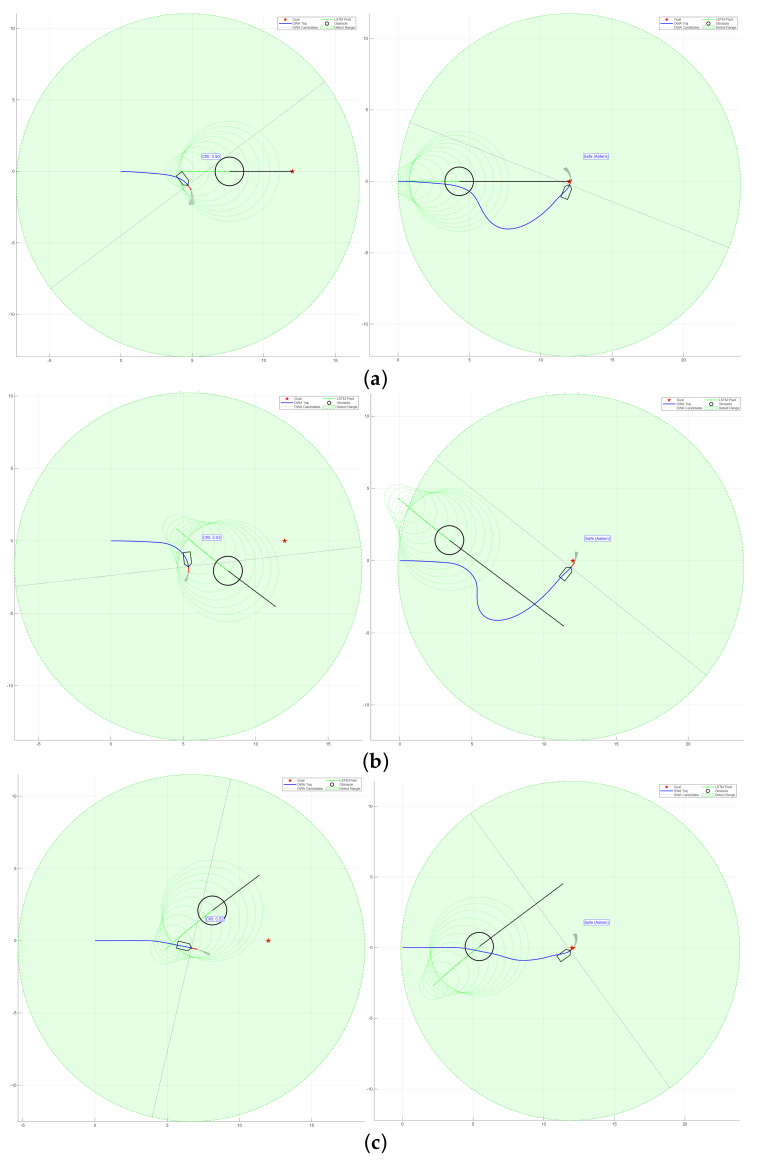
Simulation results of the DWA path planning after modification of the evaluation function based on COLREGs. (**a**) Head-on situation; (**b**) crossing situation (target on the right); (**c**) crossing situation (target on the left).

The evaluation function integrates LSTM-based perception with COLREGs-based decision-making. As shown in Figure 15, the framework uses LSTM to predict the motion of obstacles and create a dynamic risk region. At the same time, it applies COLREGs [33,34] constraints to guide the USV’s heading.

In the conventional DWA, heading is evaluated based solely on the alignment between the robot’s predicted orientation and the target goal. To ensure navigation safety in dynamic environments and strict adherence to COLREGs, an improved heading evaluation function is proposed. The heading evaluation function H(v,ω) which integrates a rule−compliance correction term into the standard goal-oriented objective.

The improved heading evaluation function is defined as follows:

(13) $$H(v,\omega )=S_{goal}(v,\omega )+\eta (d)\cdot S_{COLREGs}(v,\omega )$$

where

- $S_{goal}(v,\omega )$: the basic goal-alignment score;
- η(d): the dynamic weight for distance-dependent;
- $S_{COLREGs}(v,\omega )$: the weight for heading.

By incorporating the predicted dynamic obstacle information and the USV’s current state, this module identifies the encounter situation. Then, dynamically adjusts the heading score, imposing penalties on non-compliant maneuvers to ensure the selected trajectory adheres to maritime traffic rules.

The predicted trajectory generated by the LSTM is mapped into a dynamic danger zone, illustrated as the green dashed area in Figure 15. The evaluation function ${\mathrm{EvalDB}}_{i,k}$ is adjusted to penalize any candidate trajectory intersecting this zone, ensuring the USVs proactively avoid the obstacle’s future path.

(14) $${\mathrm{EvalDB}}_{i,k}=n\left(\frac{\omega_{1}\cdot EvalDB_{i,1}}{\sum_{j=1}^{n}EvalDB_{j,1}}+\frac{\omega_{2}\cdot EvalDB_{i,2}}{\sum_{j=1}^{n}EvalDB_{j,2}}+\frac{\omega_{3}\cdot EvalDB_{i,3}}{\sum_{j=1}^{n}EvalDB_{j,3}}\right)-\omega_{4}\cdot EvalDB_{i,4}$$

where

- ${\mathrm{EvalDB}}_{i,k}$: a collection of parameters, where i denotes the i-th parameter and k denotes the scores in EvalDB for heading, obstacle distance, and velocity, respectively; $EvalDB_{i,4}$ denotes the penalty score for proximity to the dynamic obstacle zone predicted by LSTM. This penalty appears when near dynamic obstacles, increases linearly within the zone, and is set to 0 outside the zone;
- ω: weight for each parameter;
- n: number of motion candidates.

## 5. A*-DWA Fusion

### 5.1. Algorithmic Flowchart

**Figure 16:**
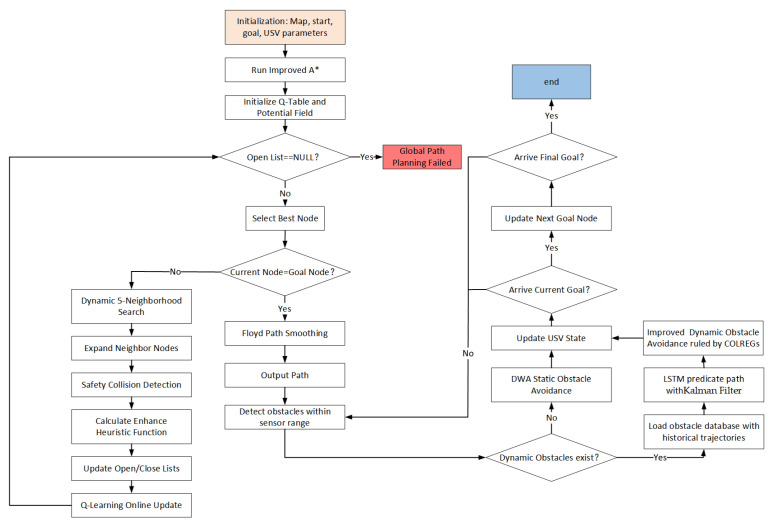
Flowchart of the improved algorithm.

We integrated both global and local path planning algorithms to propose a combined A*-DWA. The specific flowchart is illustrated in Figure 16. The algorithm enhances its global path search capability through dynamic five-neighborhood exploration and adaptive weight adjustment via reinforcement learning, while also achieving a shorter and smoother route through path optimization.

The local algorithm predicts the short-term motion trajectories of dynamic obstacles through LSTM, and by incorporating the improved DWA algorithm, it enhances predictive avoidance of obstacles, thereby improving navigation safety.

### 5.2. Simulation

**Figure 17:**
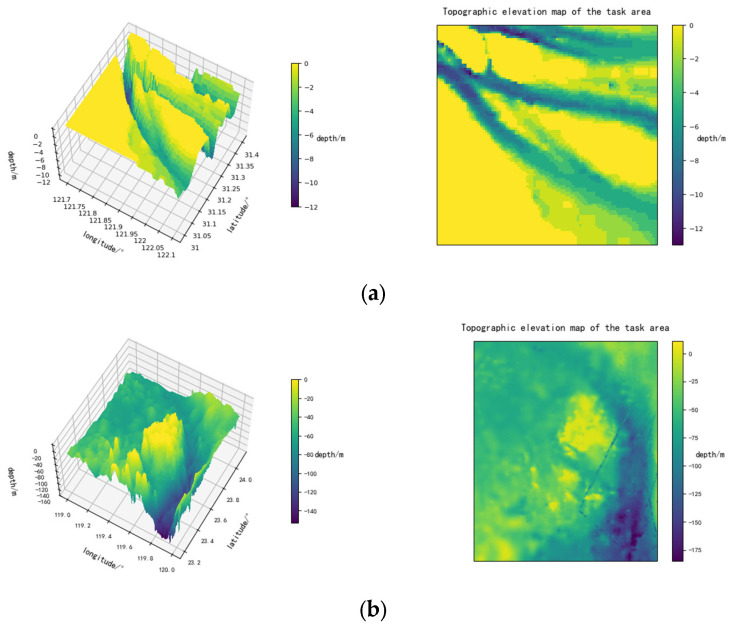
Environmental map: (**a**) Region: 31.4° N, 121.6° E to 31.0° N, 122.0° E; (**b**) region: 31.4° N, 121.6° E to 31.0° N, 122.0° E.

We conducted algorithmic simulation tests using the two maps shown in Figure 17, performing experiments in both narrow waterways and open sea areas. During initialization, a default expansion range was set to prevent failures in local path planning.

**Figure 18:**
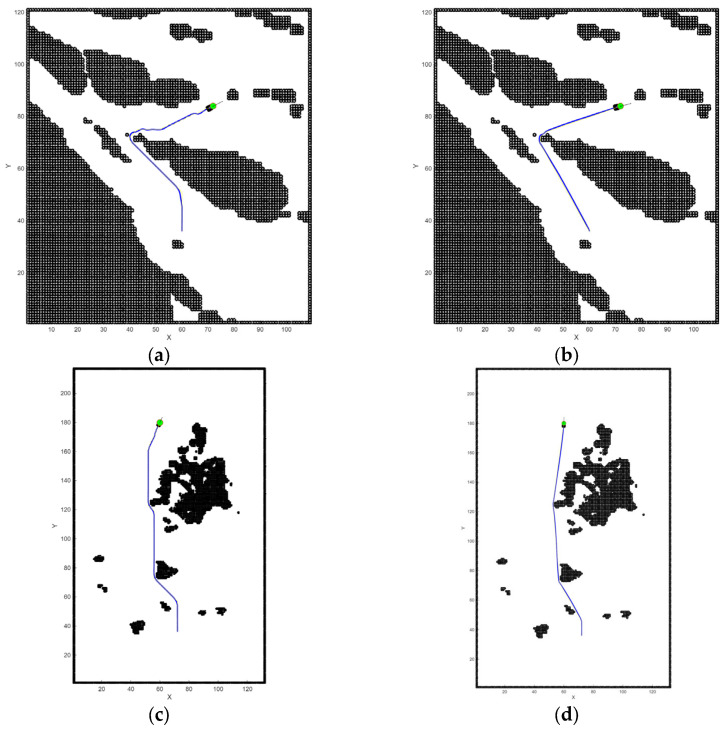
Comparative global path planning on two maps: (**a**) Narrow waters with A*; (**b**) narrow waters with Improved A*; (**c**) open waters with A*; (**d**) open waters with Improved A*.

Figure 18 and Table 8 indicate that the Aalgorithm, when enhanced with a dynamic environment layer and reinforcement learning, can reduce the number of expanded nodes and optimize the path strategy. Furthermore, the secondary optimization algorithm further enhances path smoothness and reduces the number of turns. Through the improved Aalgorithm, the SUV consumes less energy during missions, leading to higher efficiency.

**Figure 19:**
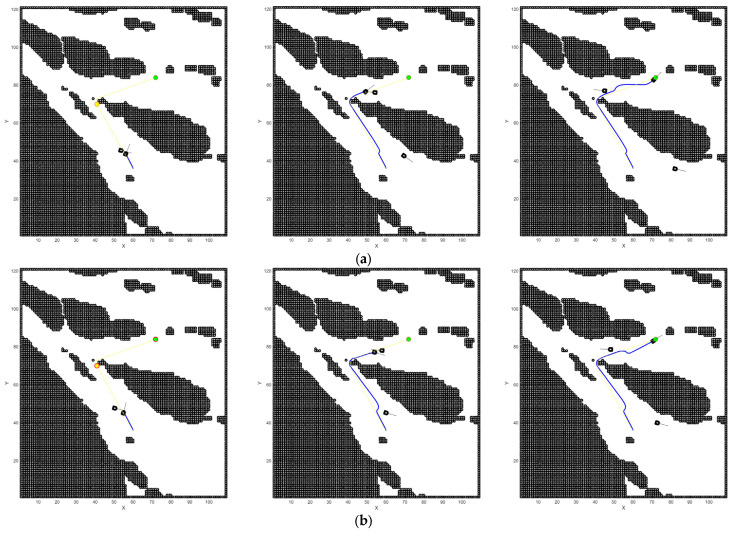
Comparative local path planning in narrow waters with dynamic obstacles. The blue line indicates the DWA trajectory tracking path, the yellow line represents the A* planned route, the yellow point denotes the current target, the green point marks the final destination, and the black areas represent obstacles. (**a**) DWA; (**b**) improved DWA.

In narrow waters, the conventional algorithm demonstrates significant limitations. It cannot anticipate the trajectory of dynamic obstacles, selecting only the optimal route for the current moment without considering the obstacle’s movement in the next instance. In Figure 19a, the conventional DWA algorithm comes to a complete stop because the updated DWA position falls within the hazard zone of a dynamic obstacle, with no feasible direction to proceed, thereby resulting in a trapped and dangerous situation. In contrast, the improved DWA, as shown in Figure 19b, is capable of predicting the trajectory of dynamic obstacles in advance and ensures that its heading does not intersect with that of the obstacles, leading to significantly better performance.

**Figure 20:**
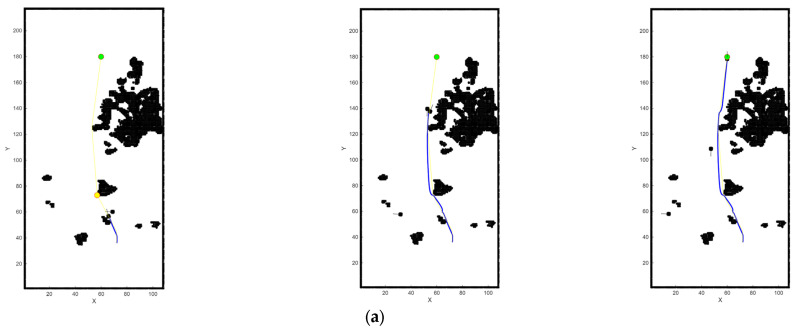

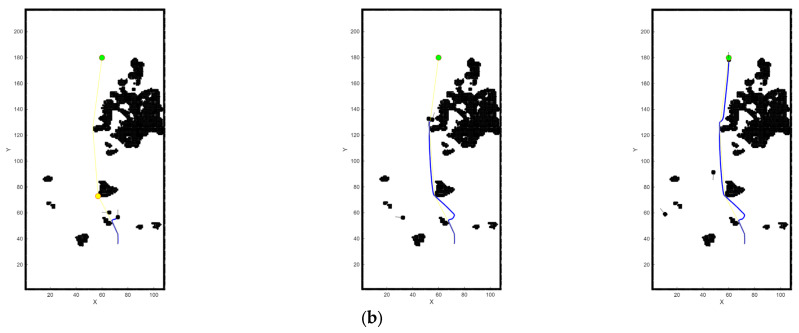
Comparative local path planning in open waters with dynamic obstacles. (**a**) DWA; (**b**) improved DWA.

In open waters shown in Figure 20, the conventional DWA algorithm, much like in narrow waters, cannot ensure the USVs remain in a relatively safe state in the next moment. Moreover, in head-on encounter situations, it fails to initiate turns proactively, which poses certain safety risks.

Through simulation comparisons, it is observed that the conventional DWA often struggles with slow planning and inaccuracies when encountering fast-moving dynamic obstacles, and may even cause the USVs to stop within hazardous areas. In contrast, the improved DWA algorithm, by incorporating LSTM-based path prediction and modifications to the evaluation function, enables the USVs to perform local obstacle avoidance proactively and safely. As a result, the improved DWA demonstrates superior obstacle-avoidance performance in the presence of dynamic obstacles, both in narrow waterways and open sea areas.

## 6. Discussion

By integrating reinforcement learning with the traditional A* algorithm, the selection of the heuristic function in A* can be enhanced. This approach reduces action consumption through a dynamic five-neighborhood search and optimizes the final path to generate the shortest and most cost-effective route. When combined with an LSTM-based path prediction algorithm, it mitigates risks posed by dynamic obstacles and improves local planning stability. This path planning algorithm is adaptable to most environments, thereby significantly enhancing the safety of USVs in complex maritime areas.

However, if compared with other hybrid planners, such as RL combined with Model Predictive Control (MPC), reveals notable distinctions in weaknesses. MPC-based solutions operate by solving optimization problems at every time step. This continuous calculation allows them to strictly handle kinematic constraints and ensure dynamic feasibility, even under environmental disturbances. Our algorithm exhibits reduced robustness against extreme dynamic disturbances when compared to RL-based methods.

Second, the current simulation environment assumes an idealized kinematic model and does not explicitly account for environmental disturbances such as strong winds, waves, and ocean currents. In real-world maritime operations, these disturbances introduce significant drift and uncertainty that can affect the USV’s global path planning and tracking precision. Although the Kalman Filter currently employed addresses sensor noise, it does not fully compensate for the complex non-linear dynamics introduced by severe sea states.

Future Work Future research will focus on incorporating a high-fidelity hydrodynamic model to simulate wind and wave effects. Additionally, we aim to explore more robust cost functions that scale with prediction uncertainty to further enhance safety in complex, stochastic environments.

## Figures and Tables

**Table 1:** Node index.

Azimuth	Reserved Node	Discard Node	Unreachable Node
337.5°~22.5°	{1,2,3,4,5}	{6,7,8}	{6,7,8}
22.5°~67.5°	{1,2,3,5,8}	{4,6,7}	{4,6,7}
67.5°~112.5°	{2,3,5,7,8}	{1,4,6}	{1,4,6}
112.5°~155.5°	{3,5,6,7,8}	{1,2,4}	{1,2,4}
155.5°~202.5°	{4,5,6,7,8}	{1,2,3}	{1,2,3}
202.5°~247.5°	{1,4,6,7,8}	{2,3,5}	{2,3,5}
247.5°~292.5°	{1,2,4,6,7}	{3,5,8}	{3,5,8}
292.5°~337.5°	{1,2,3,4,6}	{5,7,8}	{5,7,8}

**Table 2:** Detailed explanation of reward function components.

Reward Component	Mathematical Expression	Parameter Explanation	Physical Meaning
Basic Movement Cost	$r_{distance}=-d$	d: Euclidean distance of movement	Penalize long paths to encourage shortest path selection
Progress Reward	$r_{progress}=k_{p}\cdot \Delta d$	$\Delta d=dist_{now}-dist_{next}$ $k_{p}$: progress coefficient	Reward effective progress toward the goal point
Smoothness Penalty	$r_{smooth}=-k_{s}\cdot I_{diagonal}$	$I_{diagonal}$: diagonal indicator function $k_{s}$: smoothness coefficient	Apply a slight penalty to diagonal movements to enhance path smoothness
Potential Field Adjustment	$r_{potential}=-k_{pt}\cdot \Delta P$	$\Delta P=P_{next}-P_{now}$ $k_{pt}$: potential field coefficient	Adjust the path based on potential field changes to strengthen goal-directed behavior

**Table 3:** Performance comparison of the path in generated maps with Seed = 1 at various scales.

Algorithm	Path Length	Number of Turns	Smoothness	Expanded Nodes
Conventional A* (25 × 25)	39.56	11	0.22	292
Improved A* (25 × 25)	38.16	8	0.11	276
Conventional A* (50 × 50)	78.43	27	0.31	1034
Improved A* (50 × 50)	74.41	20	0.11	871
Conventional A* (75 × 75)	114.95	29	0.16	1958
Improved A* (75 × 75)	111.89	20	0.06	1536
Conventional A* (100 × 100)	157.92	51	0.25	3368
Improved A* (100 × 100)	152.28	33	0.12	2852

**Table 4:** Performance comparison of the path in generated maps with Seed = 100 at various scales.

Algorithm	Path Length	Number of Turns	Smoothness	Expanded Nodes
Conventional A* (25 × 25)	38.39	21	0.53	252
Improved A* (25 × 25)	35.68	11	0.22	211
Conventional A* (50 × 50)	78.67	24	0.24	789
Improved A* (50 × 50)	76.38	15	0.10	630
Conventional A* (75 × 75)	117.88	44	0.25	2347
Improved A* (75 × 75)	113.34	29	0.10	1876
Conventional A* (100 × 100)	155.58	51	0.27	3391
Improved A* (100 ×100)	148.30	37	0.11	2763

**Table 5:** Performance comparison of the path in generated maps with Seed = 1000 at various scales.

Algorithm	Path Length	Number of Turns	Smoothness	Expanded Nodes
Conventional A* (25 × 25)	37.80	16	0.31	218
Improved A* (25 × 25)	35.87	11	0.17	168
Conventional A* (50 × 50)	78.43	26	0.28	1021
Improved A* (50 × 50)	74.91	14	0.10	789
Conventional A* (75 × 75)	116.71	40	0.29	2018
Improved A* (75 × 75)	111.12	28	0.08	1329
Conventional A* (100 × 100)	161.10	53	0.31	4362
Improved A* (100 × 100)	155.62	38	0.17	3846

**Table 6:** LSTM Training parameters.

Parameter	Value
Input Features	Δx,Δy,sinθ,cosθ,Δθ,Trend
Hidden Layers	2 (128 units, 64 units)
Optimization Algorithm	Adam
Initial Learning Rate	0.005
Mini-Batch Size	32
Training/Validation Split	80%/20%

**Table 7:** The error between the true route and the predicted values by LSTM.

Step	Mean Error	Max Error	Min Error	Standard Deviation
10	0.156	0.378	0.009	0.107
20	0.786	1.903	0.428	0.545
30	2.0306	5.448	0.064	1.4357
40	4.027	12.083	0.274	2.795
50	6.828	22.112	0.24	4.827
60	10.511	35.097	0.46	7.658

**Table 8:** The parameters of the global path.

Path Length	Turning Points	Smoothness	Expand Nodes
81.255	22	0.8764	1680
74.295	3	0.0139	1435
155.6	20	0.0734	3681
148.64	4	0.0033	3214

## Data Availability

The data contained within the article.

## Abbreviations

The following abbreviations are used in this manuscript:

USVs	Unmanned Surface Vehicles
LSTM	Long Short-Term Memory
DWA	Dynamic Window Approach
COLREGs	Convention on the Collision Regulations for the Prevention of Collisions at Sea
KF	Kalman Filter
